# Depression Negatively Impacts Survival of Patients with Metastatic Prostate Cancer

**DOI:** 10.3390/ijerph15102148

**Published:** 2018-09-29

**Authors:** Po-Hung Lin, Jui-Ming Liu, Ren-Jun Hsu, Heng-Chang Chuang, Su-Wei Chang, See-Tong Pang, Ying-Hsu Chang, Cheng-Keng Chuang, Shun-Ku Lin

**Affiliations:** 1 Division of Urology, Department of Surgery, Chang Gung Memorial Hospital at Linkou, Taoyuan 333, Taiwan; m7587@adm.cgmh.org.tw (P.-H.L.); jacobpang@cloud.cgmh.org.tw (S.-T.P.); anatomy@cloud.cgmh.org.tw (Y.-H.C.); chuang89@cgmh.org.tw (C.-K.C.); 2 Graduate Institute of Clinical Medical Science, College of Medicine, Chang Gung University, Taoyuan 333, Taiwan; 3 Division of Urology, Department of Surgery, Taoyuan General Hospital, Ministry of Health and Welfare, Taoyuan 330, Taiwan; mento1218@gmail.com (J.-M.L.); chuang20110617@yahoo.com.tw (H.-C.C.); 4 Department of Medicine, National Yang-Ming University, Taipei 112, Taiwan; 5 Graduate Institute of Life Sciences, National Defense Medical Center, Taipei 114, Taiwan; hsurnai@gmail.com; 6 Biobank Management Center of Tri-Service General Hospital, National Defense Medical Center, Taipei 114, Taiwan; 7 Department of Pathology and Graduate Institute of Pathology and Parasitology, Tri-Service General Hospital, National Defense Medical Center, Taipei 114, Taiwan; 8 Clinical Informatics and Medical Statistics Research Center, College of Medicine, Chang Gung University, Taoyuan 333, Taiwan; shwchang@mail.cgu.edu.tw; 9 Division of Allergy, Asthma, and Rheumatology, Department of Pediatrics, Chang Gung Memorial Hospital at Linkou, 333 Taoyuan, Taiwan; 10 Department of Traditional Chinese medicine, Taipei City Hospital, Ren-Ai Branch, Taipei 106, Taiwan; 11 Institute of Public Health, National Yangming University, Taipei 112, Taiwan

**Keywords:** cancer survival, depression, metastatic prostate cancer, National Health Insurance Research Dataset

## Abstract

The prevalence of depression in patients with cancer is high, especially for patients with advanced cancer. In this study, we evaluated the prevalence of depression in prostate cancer patients in Taiwan and the association between depression and mortality in prostate cancer. This study included 1101 newly diagnosed patients with prostate cancer. We tracked the medical information of these patients from diagnosis until the end of 2012. Patients were divided into two groups according to presence or absence of depression diagnosis, and were further divided into three stages by initial treatments: localized or locally advanced, metastatic, and castration-resistant prostate cancer. Of 1101 participants, 267 (24.3%) had depression. By the end of the follow-up period (M = 8.30 ± 3.12 years), 77 (28.8%) patients in the depression group and 194 (23.3%) in the non-depressed group died. Depression was associated with higher mortality risk, (aHR 1.37; 95% CI [ 1.04–1.80]; *p* value 0.01). Patients in the metastatic prostate cancer group with depression had a significantly higher mortality risk compared to the non-depressed group, (aHR, 1.49; 95% CI [1.05–2.11]; *p* value 0.02). The impact of depression on mortality risk was not significant in either the localized or locally advanced or the castration-resistant prostate cancer groups. Our study showed that depression is related to an increased mortality risk for patients with prostate cancer, especially for metastatic prostate cancer. These results indicate that urologists should pay attention to the mood and psychiatric disorders of patients with prostate cancer.

## 1. Introduction

Cancer is one of the most prevalent diseases in modern society, impacting patients both physically and psychologically. Cancer-related symptoms and treatment complications may also increase the risk of depression. Canoui-Poitrine et al., showed that the prevalence of depression among elder patients with cancer were associated with impaired mobility and function status, inadequate social support and cancer-related pain [1]. In the contrast, being diagnosed with cancer may cause anxious and depressive symptoms in patients. The study conducted by Meyer et al., revealed that patients with advanced cancer were more likely to have major depressive episodes [2].

The prevalence of depression in patients with cancer ranges from 1.5% to 50%, and the median prevalence rates are between 22% and 29% [3]. Many studies have shown relationships between depression and different types of cancers, such as breast cancer and hepatocellular carcinoma [4,5,6]. Depressive disorder may have a negative impact to the survival of patients with cancers. A small scale randomized trial demonstrated that major depression syndrome significantly predicted worse survival in patients with metastatic non-small-cell lung cancer [7]. Another study revealed that decreasing depression symptoms over the first year were associated with longer survival among patients with metastatic breast cancer [4]. However, there is no large-scale study to investigate the association of depression disorder and cancer at all stages.

Prostate cancer is the most common urologic malignancy in males around the world. In Taiwan, it was the 5th most common cancer and the 7th cancer most likely to result in death in 2014. Additionally, the incidence of prostate cancer increased from 8.66 per 100,000 persons in 1993 to 29.22 per 100,000 persons in 2013 and the mortality rate increased from 3.28 per 100,000 patients in 1993 to 6.52 per 100,000 patients in 2013 [8]. Given this, prostate cancer is a very important public health issue in Taiwan. Besides, the clinical course of prostate cancer is slower comparing to other malignancies, and it is mainly diagnosed in elder patients. In this aspect, depression disorder may be a notable issue in patients with prostate cancer. In the present study, we aimed to conduct a population-based cohort study to investigate the prevalence rate of depression after the diagnosis of prostate cancer, and assessed the association between depression and the survival of patients with prostate cancer.

## 2. Materials and Methods

### 2.1. Database

We designed a retrospective cohort study using the Longitudinal Health Insurance Database 2000 (LHID2000), a million-person database randomly sampled from the Taiwanese National Health Insurance Research Dataset (NHIRD) for analysis. Because national health insurance in Taiwan covers more than 99% of the country’s population and most of the hospitals and clinics, it is well suited for long-term cohort research [9]. The NHIRD provides each patient’s medical diagnoses as classified using the International Classification of Diseases, 9th revision, Clinical Modification (ICD-9-CM) [10]. Therefore, the investigators could screen patients with prostate cancer and depression [11]. The institutional review board of Chang Gung Memorial Hospital reviewed the research plan and approved it (103–2084B).

### 2.2. Study Participants

**Figure 1:**
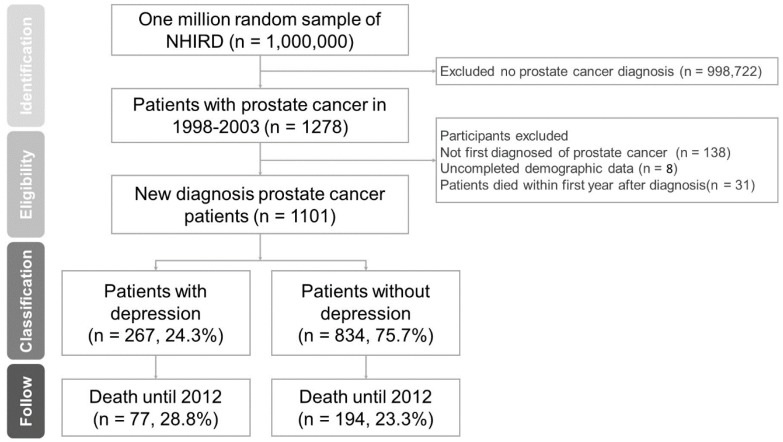
Of 1101 participants, 267 (24.3%) had depression. By the end of the follow-up period (M = 8.30 ± 3.12 years), 77 (28.8%) patients in the depression group and 194 (23.3%) in the non-depressed group died.

We collected patients with both prostate cancer diagnosis (ICD-9-CM code 185) and catastrophic illness certificates from 1998 to 2003. The government would approve the catastrophic illness certificates and remit medical expenses after reviewing the diagnosis and relevant information. Therefore, catastrophic illness certificates could be an adjunct condition for confirmation of diagnosis in a database study [12]. From 1998 to 2003, we collected information for a total of 1278 patients with prostate cancer from the database. We excluded patients who had previously been diagnosed with prostate cancer (*n* = 138), patients with incomplete data (*n* = 8), and those who had died within one year of diagnosis (*n* = 31). Ultimately, the sample included a total of 1101 newly diagnosed prostate cancer patients. We recorded all participants’ medical records from their prostate cancer diagnosis until 2012 and divided the patients into two groups according to whether or not they had a diagnosis of depression (ICD-9-CM codes 311, 296.2, 296.3, 296.5, 296.8, 300.4, 309.0, 309.1, 648.4, and 780.7) by psychiatric specialist after their prostate cancer diagnosis. Figure 1 shows the research process and the number of participants.

### 2.3. Study Outcomes

The outcome measured in this study was mortality by any cause. We collected all patients’ hospital medical information, which is recorded in the “inpatient required by admissions” file of the NHIRD. We defined the conditions of death as both the reason for discharge from hospital is death and withdrawal from health insurance. The follow-up period spanned from the day of prostate cancer diagnosis to the patients’ death or the end of the study (31 December 2012).

### 2.4. Adjustment of Covariates

The study included comorbidity covariates such as diabetes mellitus (ICD-9-CM: 250), chronic kidney disease (ICD-9-CM: 585, 586, and 588), a cerebral vascular accident (ICD-9-CM: 430–438), coronary heart disease (ICD-9-CM: 410–414), heart failure (ICD-9-CM: 428), liver cirrhosis (ICD-9-CM: 571), and hypertension (ICD-9-CM: 401–405). We analyzed the demographics of patients with prostate cancer including age (categorized into four groups: <60, 60–70, 70–80, and older than 80 years), urbanization of living area (classified into very high, high, moderate, and low urbanization), and the amount the participant was insured for, which was related to their income. We assessed the above factors for the distribution of depression and corrected for the mortality risk.

We used the initial treatment to evaluate the clinical stage of prostate cancer because the NHIRD did not contain clinical stage of prostate cancer. The localized or locally advanced prostate cancer group included patients who were undergoing radical prostatectomy or radiation therapy, the metastatic prostate cancer group included patients who initially received androgen deprivation therapy (ADT).

### 2.5. Statistical Methods

We used Chi-square test to analyze the factors associated with depression in patients with prostate cancer, including demographic factors and comorbidities. We applied the Cox proportional regression model to assess the effect of depression on the mortality risk in patients with prostate cancer. We set the significance level (α) to 0.05, and the control variables included age at diagnosis, urbanization of living area, amount of health care insurance, and comorbidities. We performed adjusted hazard ratios (aHRs) accompanied by 95% confidence intervals (95% CI) to represent the relative mortality risk. We present the fraction of living patients in the follow-up period after diagnosis with Kaplan-Meier curves and log-rank tests. We used the statistical software package SAS (version 9.4, SAS Institute, Cary, North Carolina, United States of America) for data analysis and applied MedCalc statistical software (version 16.8.4, MedCalc Software bvba, Ostend, Belgium) to plot survival curves.

## 3. Results

In the present study, a total of 1101 newly diagnosed patients with prostate cancer were enrolled from the NHIRD between 1998 and 2003. Among patients with prostate cancer, 267 suffered from depression (24.3%) while 834 did not (75.7%). The mean follow-up period was 8.30 ± 3.12 years. At the end of 2012, 77 in the depression group had died, while 194 had died in the non-depression group (Figure 1). The demographic characteristics are listed in Table 1. There were no statistically significant differences in age, urbanization, or insurance amount between the depression and non-depression groups. Patients with depression had more comorbidities (28.8%) than patients without depression (23.3%). The prostate cancer stage distribution was different between the depression and non-depression groups (*p* = 0.04); the percentage of metastatic prostate cancer was 70.8% in the group with depression and 60.0% in non-depression group (Table 1).

Table 2 shows the Cox regression analysis results for the independent risk factors associated with prostate cancer mortality. After adjusting for age, income, urbanization, and comorbidities, depression (aHR 1.37; 95% CI [1.04–1.80]; *p* value 0.01), hypertension (aHR 1.61; 95% CI [1.23–2.10]; *p* value 0.01), and liver cirrhosis (aHR 1.30; 95% CI [1.00–1.69]; *p* value 0.05) were statistically significant associated with prostate cancer mortality. For the different distribution of comorbidities, ages, and socioeconomic status, we further used the odds ratio of logistic regression as a weight to re-calculate the results. The higher risk of death in patients with depression was still significant after correction (aHR 1.68; 95% CI [1.02–2.14]; the risk of death from metastatic prostate cancer group also significantly increased (aHR, 1.58; 95% CI [1.05–2.43]).

The association between mortality and different prostate cancer groups using Cox regression model is shown in Table 3. Patients with depression in the metastatic prostate cancer group had a significant higher mortality risk compared to patients without depression (aHR, 1.49; 95% CI [1.05–2.11]; *p* = 0.02). The mortality risks were not statistically significant in the localized or locally advanced prostate cancer groups.

**Figure 2:**
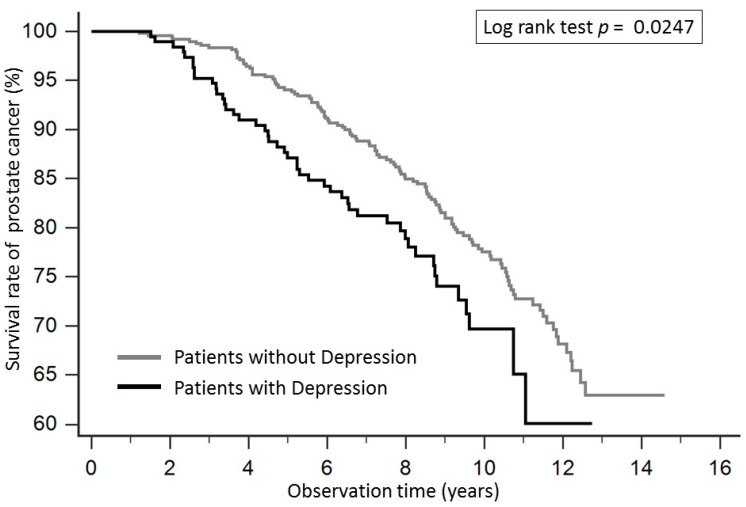
The Kaplan-Meier survival curve for patients with prostate cancer according to depression. The survival rate in patients with depression was significantly lower compared to those without depression, and the *p* value of the log-rank test was 0.0247.

**Figure 3:**
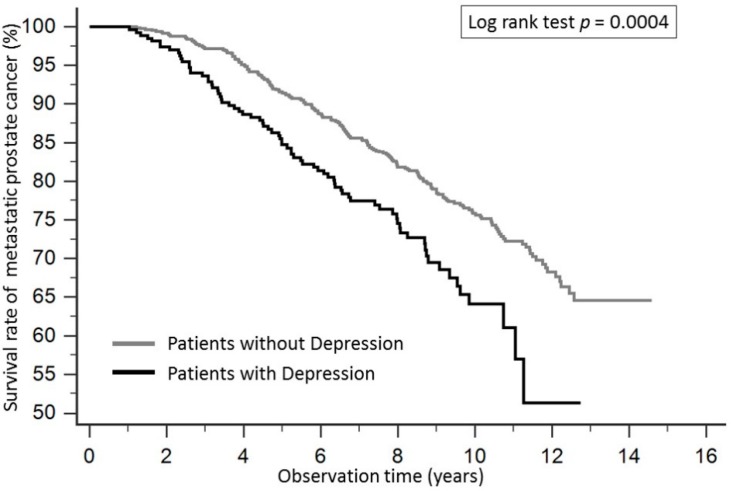
The Kaplan-Meier survival curve for patients with metastatic prostate cancer according to depression. Patients with depression in the metastatic prostate cancer group had significantly lower survival rates compared with patients without depression, and the *p* value of log-rank test was 0.0004.

The Kaplan-Meier survival curve for patients with prostate cancer according to depression is shown in Figure 2. The survival rate in patients with depression was significantly lower compared to those without depression, and the *p* value of the log-rank test was 0.0247. Patients with depression in the metastatic prostate cancer group also had significantly lower survival rates compared with patients without depression, and the *p* value of log-rank test was 0.00004 (Figure 3).

## 4. Discussion

In the present study, we used the Taiwanese NHIRD to evaluate the impact of depression, which was diagnosed after cancer, on the survival of patients with prostate cancer. Among 1101 enrolled prostate cancer patients, 267 (24.3%) patients developed depression. For the patients who had developed depression, 70.8% were metastatic prostate cancer patients. Patients with depression had higher mortality rates compared to patients without depression (aHR = 1.37, *p* = 0.01). Further subgroup analysis showed that the main negative impact of depression on survival was for the group with metastatic prostate cancer (aHR = 1.49, *p* = 0.02). This result indicates that patients with metastatic prostate cancer easily develop depression, and they may have a 49% higher risk of mortality compared to patients in the same disease stage without depression.

The prevalence of depression in prostate cancer patients is relatively high. Krebber et al., conducted a meta-analysis of diagnostic interviews and self-report instruments to evaluate the prevalence of depression in patients with cancer [13]. The prevalence of depression was 14% as measured by diagnostic interviews and 27% as measured by self-report instruments during treatment. One year after diagnosis, the prevalence of depression was 8% and 15%, respectively. In our study, we used the ICD-9 code of depression as the diagnostic criteria. This may have led to an over-estimation of the prevalence, but our prevalence result (24% of the sample) was comparable to previous literature. 

We divided the prostate cancer into three groups according to clinical stage and severity of disease: localized or locally advanced prostate cancer and metastatic prostate cancer according to the initial treatment after cancer diagnosis. Among the patients who developed depression after prostate cancer was diagnosed, 70% were patients in the metastatic prostate cancer group. Indeed, when patients are diagnosed with advanced or metastatic disease, they will experience great stress. Meyer et al., evaluated the risks of a major depression episode and found that advanced stage cancer patients were more likely to have an initial major depression episode (Odds ratio = 27.3, 95% CI [14.8–50.4], *p* < 0.001) [2].

In the multivariate analysis, depression was an independent factor that negatively impacted the survival of patients with prostate cancer. This finding was similar to that reported in the previous literature. A meta-analysis conducted by Satin et al., revealed that depression predicted mortality, but not disease progression in patients with cancer [14]. We further analyzed the effect of depression on mortality in different stages of prostate cancer, and patients with metastatic prostate cancer who developed depression had a nearly 50% higher risk of mortality compared to patients without depression in the same stage. This may relate to the initial treatment for metastatic prostate cancer, namely ADT.

Since the introduction of ADT for prostate cancer by Huggins in 1941 [15], it has become the first line of treatment for patients with metastatic prostate cancer. ADT has been used as a combination therapy with radiotherapy for patients with high risk localized prostate cancer, salvage therapy for patients with a positive surgical margin after radical prostatectomy, and an alternative treatment for patients with poor performance status [16]. One study revealed that the use of ADT increased from 1990s to 2000 and the total use rate of ADT in prostate cancer between 2000 and 2002 was around 44% [17,18].

In recent years, many studies have been conducted to explore the association between ADT and depression. Treatment with ADT in patients with prostate cancer was significantly associated with increased depression compared to patients with prostate cancer who were not treated with ADT [19,20]. Another study also showed that the rate of clinically significant depressive symptomatology in patients who received ADT for prostate cancer was higher than patients without cancer [21]. Depression was found to be associated with increased mortality of patients with prostate cancer [22], although that study considered depression diagnosed before prostate cancer. There have been no studies investigating the association between depression, which was diagnosed after prostate cancer, and survival of patients with prostate cancer. Our study has shown that depression diagnosed after cancer was associated with decreased survival of patients with metastatic prostate cancer. Depression is associated with an increase in pro-inflammatory cytokines [23]. Depression is associated with poor prognosis of cancer and this association is due to increase in cytokine production [24]. New treatment strategy should include the prescription of antidepressants to depressed prostate cancer patients because antidepressant such as fluoxetine was found to reduce pro-inflammatory cytokines in an animal model of depression [25]. Antidepressants may improve the prognosis of cancer by reducing the severity of depression.

Due to the cultural characteristics of Asian people, patients with cancers are less willing to disclose their condition and seek positive social assistance. Patients often suffer from the symptoms and side effects of treatments alone, and inadequate support leading to more severe depression [26]. Past research has revealed that family support is associated with emotional-focused coping and good quality of life [27]. Also, exercise intervention, intensive nutrition, and stress-relief practices have a significant effect on the melancholy of cancer patients [28]. Based on these findings, more concern about the depressive conditions in patients with cancer and increased mental care resources are recommended for the health care institutions and governments.

### Study Limitations

Our study has some limitations. First, we used the ICD-9 code as the criteria for depression because detailed diagnostic interviews or self-report instrument results were not available in the database. In this setting, over-estimation of depression may have led to bias in our study. Second, the details of cancer stages and grades, such as prostate specific antigen level, Gleason score, and actual tumor stage were not available in the database. As such, we used the initial treatment category of prostate cancer to surmise the stage of cancer. There would be some inter-individual difference of disease even at the same stage that may affect the outcome of prostate cancer. Third, treatments of depression, either medical or psychological, were not evaluated. The comparison of mortality between patients receiving treatment for depression and those not receiving treatment could not be conducted. Fourth, the socioeconomic status (SES) of the participants were associated with both depression and mortality. We adjusted the amount of insurance, which can roughly represent the household income of the participants and the degree of urbanization in the insured location. However, we couldn’t obtain the educational level of participants and detailed employment information from NHIRD.

## 5. Conclusions

The presented study showed that the prevalence of depression after prostate cancer was diagnosed was high. Patients who were diagnosed with metastatic prostate cancer initially and developed depression thereafter had significant higher mortality rates. These results indicate that urologist should pay attention to the mood and psychiatric disorders of prostate cancer patients. Treatment for psychiatric disorder and cancer are both important.

## Figures and Tables

**Table 1:** Demographic and medical characteristics of prostate cancer patients according to depression.

Variables	Patients with Depression No. (%)	Patients without Depression No. (%)	Chi-Square Test *p*-Value
Total	267 (100%)	834 (100%)	
Age at diagnosis			0.58
<60	14 (5.24%)	60 (7.19%)	
60–70	34 (12.73%)	142 (17.03%)	
70–80	78 (29.21%)	242 (29.02%)	
≥80	141 (52.81%)	390 (46.76%	
Urbanization			0.58
Very high	143 (53.56%)	473 (56.71%)	
High	57 (21.35%)	177 (21.22%)	
Moderate	47 (17.60%)	138 (16.55%)	
Low	20 (7.49%)	46 (5.52%)	
Insured amount (NT$) ^a^			0.43
Dependent	59 (22.10%)	216 (25.90%)	
1–199,99	114 (42.70%)	314 (37.65%)	
20,000–39,999	61 (22.85%)	165 (19.78%)	
≥40,000	33 (12.36%)	139 (16.67%)	
Comorbidity			
Diabetes mellitus	134 (50.19%)	348 (41.73%)	0.04
Chronic kidney disease	74 (27.72%)	224 (26.86%)	0.06
Cerebrovascular accident	152 (56.93%)	355 (42.57%)	0.66
Coronary heart disease	170 (63.67%)	433 (51.92%)	0.04
Heart failure	90 (33.71%)	184 (22.06%)	<0.001
Liver cirrhosis	139 (52.06%)	295 (35.37%)	0.02
Hypertension	232 (86.89%)	619 (74.22%)	0.03
Prostate cancer type			0.04
Localize or Locally Advance	73 (27.3%)	320 (38.4%)	
Metastatic	194 (72.7%)	51 4 (61.7%)	

^a^ NT$ represent New Taiwan dollars, of which 1 US $ = 31.1 NT$.

**Table 2:** Adjusted hazard ratio with 95% confidence interval of the mortality in national prostate cancer cohort.

Variables	Adjust Hazard Ratio	95% Confidence Interval	*p*-Value
Depression			
Non depression	[Reference]	[Reference]	[Reference]
1.37	1.04-1.80	0.01	1.37
Age at diagnosis			
<60	[Reference]	[Reference]	[Reference]
60–70	0.95	0.38–2.37	0.91
70–80	1.24	0.55–2.81	0.61
≥80	1.63	0.73–3.64	0.23
Urbanization			
Very high	[Reference]	[Reference]	[Reference]
High	1.29	0.95–1.75	0.11
Moderate	1.73	1.26–2.38	0.02
Low	1.61	0.95–2.73	0.08
Insured amount (NT$) ^b^			
Dependent	[Reference]	[Reference]	[Reference]
1–19,999	1.25	0.93–1.68	0.13
20,000–39,999	0.83	0.62–0.95	0.03
≥40,000	0.57	0.39–0.71	0.02
Comorbidity			
Diabetes mellitus	1.19	0.93–1.52	0.18
Chronic kidney disease	1.08	0.80–1.46	0.61
Cerebrovascular accident	1.09	0.84–1.42	0.50
Coronary heart disease	1.15	0.77–1.70	0.50
Liver cirrhosis	1.30	1.00–1.69	0.05
Hypertension	1.61	1.23–2.10	0.01

^b^ NT$ represent New Taiwan Dollars, of which 1 US $ = 30 NT$. The hazard ratio was compared with [Reference], as hazard ratio of [Reference] = 1.

**Table 3:** Mortality in national prostate cancer cohort, analyzed by multivariable cox proportional hazards regression model and 95% confidence intervals.

Prostate Cancer Type	Adjust Hazard Ratio	95% Confidence Interval	*p*-Value
Localize or Locally Advance Prostate Cancer	1.20	0.83–2.34	0.07
Metastatic Prostate Cancer	1.52	1.04–2.10	0.02
